# The crisis of international education students and responsive service in and after COVID-19 pandemic

**DOI:** 10.3389/fpsyg.2022.1053512

**Published:** 2022-11-21

**Authors:** Li-Jie Du

**Affiliations:** Department of Sociology, College of Philosophy, Law and Politics, Shanghai Normal University, Shanghai, China

**Keywords:** the crisis, responsive service, international education students, COVID-19 pandemic, mental health

## Introduction

The COVID-19 Pandemic is still rampant around the world. In some countries, there have been second, third-wave, or even more outbreaks. The global outbreak of the COVID-19 Pandemic has caused many social problems, leaving many vulnerable people in a state of crisis around the world until now. One of the most vulnerable populations suffering from the COVID-19, ignored by social policy makers and social services providers, is international students (Baloran, 2020). Physical and psychological outcomes of COVID-19 among vulnerable populations are key elements of public health (Kashiko, 2022). The impact of COVID-19 pandemic on mental health became a special issue for many publications. As for the international students' crisis and responsive service affected by the pandemic, there have also been many discussions and studies on this topic.

## Subsections relevant for the subject

The research findings were obtained based on the following three themes: Mental health challenges for young students worldwide; The academic difficulties caused by online education; The responsive service and policies. The following is a commentary on them.

In response to the COVID-19 outbreaks, most countries or regions have taken numerous measures to impede block transit, or restricted entry and exit, and imposed blockades in some cities from the beginning of 2020. The harmful consequences of the pandemic have been reported, influencing not only economic development, international politics, social policies, health care, and educational approaches, but also severely affecting the daily lives of individuals. A variety of pandemic mitigation strategies such as social alienation, university closures, and distance learning brought mental health challenges for college students worldwide (Kim et al., 2022).

Though the cross-sectional survey which is conducted in spring 2020 that included 91,871 students from 23 countries, the result shows that Depressive symptoms were more frequent among students who suffered financial loss during the pandemic. Minimally and maximally adjusted models showed a 35% higher prevalence of depressive symptoms in students who lost economic resources compared to students with stable economic resources during the lockdown. No substantial differences in the association between students' financial loss and depressive symptoms were found across countries (Tancredi et al., 2022). International students often pay several times more in tuition than local students to complete their studies in the host country, which requires financial support from their families or the government. It was unavoidable that the academic and living circumstances of international students were affected by the economic decline during the pandemic, which also caused depression and mental health problems among students. The disruptions caused by the COVID-19 pandemic on educational and occupational opportunities may have increased their mental health vulnerability (Kim et al., 2022).

There has been much research and discussion on the academic difficulties of international and local students in and after COVID-19, which are mainly caused by school closures and online teaching. Universities in China have transitioned to online education in response to the pandemic since March 2020. After that, online teaching became conventional reactions to school closures or the permanent prevention and control measures due to sudden outbreaks of epidemic in some regions. It intensified the focus on collaboration in online learning, and encourages educators or teachers to reconsider how new forms of practice and pedagogical theory can be more effectively interwoven into teacher training (Lei and Medwell, 2021). Teacher's instruction and student's learning often take place through computers, laptops, and cell phones with Internet connections. The report finds that student-instructor and student-student interactions do not fully establish a cognitive social presence and an affective social presence (Wut and Xu, 2021). Teachers should add classroom encouragement, assessment incentives, group discussions, and participation techniques to increase the effectiveness of online instruction. In addition, universities should further increase satisfaction with online education by developing easy-to-use online courses, focusing on the functionality of electronic devices frequently used by university students, and continuously providing training and advice that can improve students' perceived utility of online courses (Han and Sa, 2021). The coping techniques of online learning are valid mostly for local students, but the problems of international students are very complicated, and the dilemma caused by the COVID-19 for them cannot be solved by improving educational technology alone. Studying abroad is not only about learning knowledge and skills from online teaching, but also a life-long journey to experience a foreign culture and social system personally, so the international students need more non-academic supportive services. For example, the ISS (International Student Services) office in some American universities responded by providing both academic and non-academic supportive programs to international students in and after the COVID-19 pandemic (Yovana and Ravichandran, 2021).

The discussion on international students is not only in education but also in other fields, such as social policy and international social work. Social work professionals can play a key role in assisting international students with cultural adaptation and social adjustment, as colleges and universities are one of the places where social work is settled, such as providing counseling services and assisting students with academic difficulties and adjustment issues (Gibelman, 2005). The key to international students achieving their own educational goals is the ability of each person to successfully adjust to the host country. Fostering a healthy multicultural environment in higher education is also important for the adjustment, where international students from different countries can live in harmony and learn happily in host countries. Moreover, supporting the adjustment of international students, promoting social diversity and empowerment are also consistent with core values of the social work profession (Park et al., 2017).

The medical social workers and psychologists in the hospital aimed to analyse the psychological status and its influencing factors of the quarantined Chinese students after they're getting off the international flights through the study at a hotel in Guangzhou, as to provide a reference for further improvement of psychological prevention and treatment strategies for the students at the quarantine places (Deng et al., 2021). The researchers have also discussed “international social work” in the articles that it has become a new topic for Chinese social work and some groups of people were concerned by international social work that has been addressed: Chinese students and visiting scholars staying overseas countries, quarantined people on international flights at the gates of entry cities, young students and their parents on charter flights back to China, residents of global communities, etc. (Du, 2021). Since March 23 of 2020, Shanghai has undertaken most of the quarantine works as the “first point of entry” for passengers of international flights to China. The Shanghai Civil Affairs Bureau activated the Shanghai Social Work Emergency Service Team to provide professional services for young students returning home and their parents who were waiting (Du and Lu, 2021), which illustrated the services provided for Chinese students returning from overseas, but did not address foreign students in China.

International students who are considered non-permanent residents are excluded from the relief programs of their host country governments. Several studies have confirmed that international students, especially those from undeveloped countries, already experience challenging life circumstances, including financial crises, in their study destinations (David, 2020). There has a large number of international students from undeveloped African countries in China. As for them, it would have been even worse after they went back to their homeland. For example, in the Central African Republic (CAR), the political unrest, a weak health system, internal poverty, and many other factors have left the country ill-prepared for a COVID-19 pandemic, resulting in a greater threat to the lives and health of the population throughout the country. Therefore, rapid measures are to be taken to prevent the further spread of COVID-19. The help from the international community is urgently needed (Gou et al., 2022). During and after the COVID-19 Pandemic, social workers have always been at the forefront of society, responding quickly and using their professionalism and strength to serve the people when a crisis occurs, so the role of “International Social Work Community Cooperation” is valued (Yuen-Tsang, 2020).

## Discussion

Although the above crises are wide-spread among young students worldwide, the dilemmas of the international student community are more highlighted and difficult to resolve because of their more complex sociocultural backgrounds. COVID-19 has added another layer of complexity for international students as they grapple with newly introduced immigration policies and compliance standards, health and safety concerns, and travel issues. International students hold temporary legal status in their host country, and the distance that exists between them and their families back home often leads to acculturative stress, which is exacerbated at a time of crisis (Yovana and Ravichandran, 2021). When teaching and learning approaches shift from offline to online, international students may take time to adapt to their new digital environment on campus, which may be very different than the sources of online information they relied on while in their home country (Chang and Gomes, 2017). Perhaps more importantly, they make invaluable intellectual, cultural, and economic contributions to their host institution and country, serving as a key aspect for advancing internationalization, inclusivity, and diversity efforts on campus (Smith, 2020), but they do not have citizenship or permanent resident status of the host country and excluded from local government welfare and relief programs, no access to get the assistance and benefits in the event of pandemic. So providing effective assistance for international students is needed.

This requires more responsibility from the higher education sector in each country. There is no doubt that non-local students have contributed immensely to the sustaining of the global higher education system. Not only do they enrich cultural diversity and enhance cultural development, but their contribution to knowledge production and innovation cannot be overstated. Therefore, promoting their well-being, especially in times of crisis, should be paramount within the global higher education sector (Amoah and Mok, 2022). Beyond that, Social work can play a major role in the mitigation of the pandemic's impact on international students, as it is a human rights profession rooted in its principles of social justice. Social work practice is performed at all three levels: the micro, mezzo, and macro (David, 2020). It attempts to propose some responding strategies from three perspectives: individual, community and government, corresponding to each of the three levels in the conclusion, which are the two active implementations relevant to the international students: the services provided by the university or school and the policies promoted by the government. The pandemic has not only significantly decreased international student mobility but is also shifting the mobility flow (Mok et al., 2021).

## Conclusion

Although international students were in a state of crisis, such as mental health challenges and the dilemma associated with online teaching, they are often ignored in some discussions about education and COVID-19-related topics. More research and findings are needed on the negative impact of the outbreak on international students in the future. It is also important that develop effective support which works for students from different socio-economic backgrounds during the COVID-19 crisis (Raaper and Brown, 2020). These students play an important role in enhancing the internationalization of the country's higher education and strengthening international communication. We call on providing better support not only for the sake of international students but also for the benefit of the international education sector (Nguyen and Balakrishnan, 2020). The negative impact of the COVID-19 on international students will remain for long time and still require interdisciplinary teamwork and international cooperation among governments.

## Author contributions

The author confirms being the sole contributor of this work and has approved it for publication.

## Funding

This article was supported by the National Social Science Fund of China under the project-A study of the transmission patterns and development paths of Chinese culture among new Chinese immigrants in Canada (Project No. 22BMZ141).

## Conflict of interest

The author declares that the research was conducted in the absence of any commercial or financial relationships that could be construed as a potential conflict of interest.

## Publisher's note

All claims expressed in this article are solely those of the authors and do not necessarily represent those of their affiliated organizations, or those of the publisher, the editors and the reviewers. Any product that may be evaluated in this article, or claim that may be made by its manufacturer, is not guaranteed or endorsed by the publisher.
